# The genomes of a monogenic fly: views of primitive sex chromosomes

**DOI:** 10.1038/s41598-020-72880-0

**Published:** 2020-09-25

**Authors:** Anne A. Andere, Meaghan L. Pimsler, Aaron M. Tarone, Christine J. Picard

**Affiliations:** 1 Department of Biology, Indiana University- Purdue University Indianapolis, Indianapolis, IN USA; 2 Department of Biological Sciences, The University of Alabama, Tuscaloosa, AL USA; 3 Department of Entomology, Texas A&M University, College Station, TX USA

**Keywords:** Entomology, Sexual selection

## Abstract

The production of male and female offspring is often determined by the presence of specific sex chromosomes which control sex-specific expression, and sex chromosomes evolve through reduced recombination and specialized gene content. Here we present the genomes of *Chrysomya rufifacies*, a monogenic blow fly (females produce female or male offspring, exclusively) by separately sequencing and assembling each type of female and the male. The genomes (> 25X coverage) do not appear to have any sex-linked Muller F elements (typical for many Diptera) and exhibit little differentiation between groups supporting the morphological assessments of *C. rufifacies* homomorphic chromosomes. Males in this species are associated with a unimodal coverage distribution while females exhibit bimodal coverage distributions, suggesting a potential difference in genomic architecture. The presence of the individual-sex draft genomes herein provides new clues regarding the origination and evolution of the diverse sex-determining mechanisms observed within Diptera. Additional genomic analysis of sex chromosomes and sex-determining genes of other blow flies will allow a refined evolutionary understanding of how flies with a typical X/Y heterogametic amphogeny (male and female offspring in similar ratios) sex determination systems evolved into one with a dominant factor that results in single sex progeny in a chromosomally monomorphic system.

## Introduction

Animals and plants exhibit typical patterns of sex chromosomes evolution in heteromorphic chromosomal systems^1^. An autosome first begins to differentiate following the acquisition of a sex-determining locus and this differentiation is maintained via reduced recombination. Eventually, this can lead to initial expansion and eventual degeneration of the Y chromosome in X/Y systems, and similar processes happening in Z/W systems^1–6^. Evolutionary theory postulates that differentiated sex chromosomes trace their ancestry to an undifferentiated autosomal pair where one of the autosomal homologs acquired a sex-determining gene and consequently sexually antagonistic mutations arose causing reduced or eliminated recombination between the pair^7,8^. Restricted recombination led to the emergence of the sex-limited chromosome, in the case of flies—usually the Y chromosome. The newly evolved sex chromosomes therefore diverge functionally and morphologically resulting in heteromorphic chromosomes^7–9^. In general, Y chromosomes contain very little genic material and the chromosome is mostly heterochromatic, typically due to the result of mutations, insertions and deletions, and transposable element activity. Of course, with every rule come the exceptions. In Diptera, the model species *Drosophila melanogaster* has heteromorphic sex chromosomes, however the ancestral Dipteran sex chromosome thought to be the dot or 4th chromosome, is an autosome in *D. melanogaster*. Furthermore, the *D. melanogaster* mode of sex determination does not depend on the presence of a male-determining locus on the Y chromosome, but rather dosage differences of genes on the X chromosome results in alternatively spliced transcripts driving the development towards either a male or female fate. Furthermore, fundamental differences in sex determination processes vary across Diptera (for a review see:^10^). For example, the mosquito *Aedes aegypti*, the house fly (*Musca domestica*) and Mediterranean fruit fly (*Ceratitis capitata*) all harbor a male determining factor present on the Y chromosome, following typical, non-Drosophilid tradition^11^. In contrast, sex determination in sciarid flies, such as *Sciara ocellaris*, relies upon dosage compensation affected by temperature-dependent paternally donated X-chromosome destruction^12,13^.

Signatures in the genome left behind from multiple evolutionary events can be used to decode the mystery of sex-determining systems in many living organisms^14–17^. Transitions of sex determination mechanisms have been found to be frequent in nature among species which display homomorphic sex chromosomes in both sexes^18^. For example, in amphibians and reptilians the turnover rate of sex-determining genes and sex chromosomes is high. Approximately 96% of amphibian species possess homomorphic sex chromosomes with a sex-determining gene that is easily and rapidly replaced by another gene of a different chromosome across their phylogeny^19–21^. Epigenetic and environmental factors such as temperature can also play a role in sex determination^22^. In comparison, species with heteromorphic sex chromosomes (XY and ZW systems) are presumed to be highly differentiated and have reached an evolutionary end point with the sex-determining gene in the sex chromosome limited sex^8,23^.

For most calyptrate flies, the common sex chromosome system is the XX/XY system^15,24^ with a homogametic female XX and a heterogametic male XY, with the sex chromosomes making up one of the six chromosome pairs. Heteromorphic sex chromosomes are observed in a majority of blow fly species (Diptera: Calliphoridae), with differentiated X and Y sex chromosomes in both morphology and sequence. Several blow fly species in the subfamily Chrysomyniae such as *Cochliomyia hominivorax, Cochliomyia macellaria, Protophormia terranovae, Phormia regina*, *Chrysomya megacephala*^25–27^ have heteromorphic sex chromosomes and sex development in most blow flies is controlled via a dominant male-determining factor on the Y chromosome^15,25,28–30^. In other blow flies such as the Lucilinae, some *Lucilia* species have significant differences in genome sizes between the sexes, which can be > 50 Mb, representing > 7% of female genomic content^31^.

**Figure 1:**
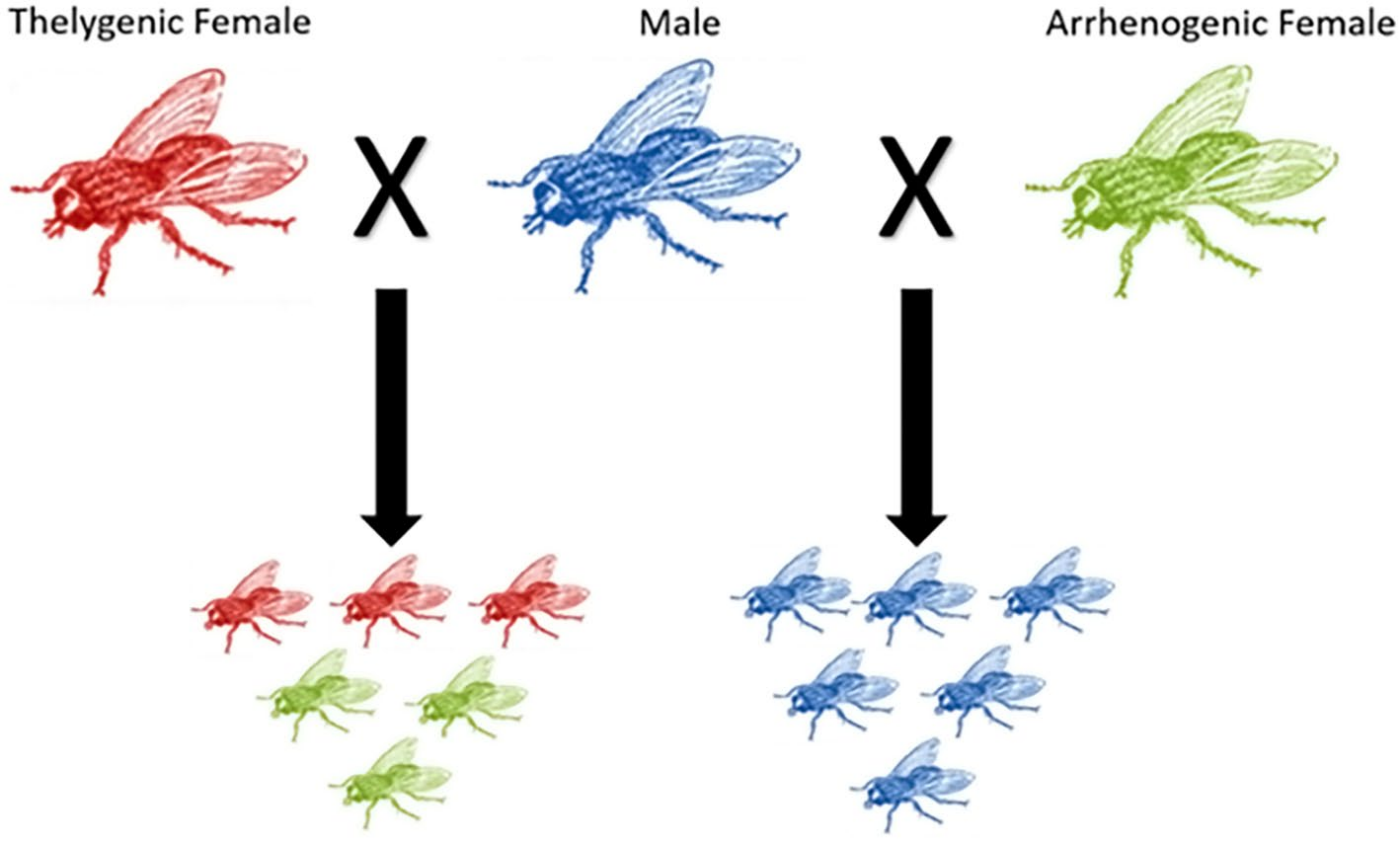
Sex determination of C. rufifacies offspring is determined by the maternal genotype. Thelygenic females produce only female offpsring while arrhenogenic females produce only male offspring. Females in the above figure are represented by the red and green colors, whereas males are blue.

However, two Chrysomyinae species *Chrysomya rufifacies* and *Chrysomya albiceps* have homomorphic sex chromosomes and both sexes have the same size genomes^25,32–35^. Furthermore, in these monogenic species, the females produce either all female or all male progeny^34,36,37^ (Fig. 1)—a divergence from the heteromorphic (differentiated) and amphogenic sex chromosome system observed in other Calliphoridae^25,38^. The genetic basis of monogeny in *C. rufifacies* has been hypothesized from mating studies, ovary and pole-cell transplantation and patterns of protein expression^36,37,39,40^: female producing flies (thelygenic females) are heterozygous for a dominant female-determiner (F/f) with predetermined sex-determining properties while the male producing females (arrhenogenic females) and males are homozygous for the recessive allele (f/f) at this same locus. Sex determination in *C. rufifacies* is largely genetic and independent of environmental factors such as diet, season and temperature^34^. However, the molecular nature of the primary sex-determining gene(s) or locus in *C. rufifacies* remains unknown.

In this study, we present the genomic sequences and the assembled genomes of male, thelygenic female, and arrhenogenic female *C. rufifacies* for the first time. We characterize putative sex chromosomes and document candidate sequences which belong to the dipteran ancestral sex chromosome (Muller F). We also show genomic evidence that these putative sex chromosomes appear to be undifferentiated, unless differentiation occurs through copy number or through small portions of the genome. These results will allow for a greater depth of evolutionary study on sex chromosomes across the Calliphorid species and give insight into the unique sex-determining mechanism of a monogenic fly.

## Results and discussion

### Sequencing and de novo genome assembly

Three separate genomes (male: M, thelygenic female: TF, and arrhenogenic female: AF) were paired-end sequenced resulting in an average read length of 100 bp and average quality score of 37 following adapter sequence trimming, low quality read filtering and overlapping pairs merged. Approximately 0.07% (M), 0.06% (TF) and 0.11% (AF) of reads were removed as they were identified as either non-fly or mitochondrial reads, resulting in 8.5 X 10^7^ (M), 1.02 X 10^8^ (TF), and 1.34 X 10^8^ (AF) high-quality reads used to assemble three genomes. Initial draft genomes were further scaffolded using the TF *C. rufifacies* transcriptome as a guide^41^. Approximately 95% of the reads from each sex type mapped back to the contigs with an average coverage range of 27–42X reads suggesting that most of the reads were utilized in the genome construction (Table 1). From a set of 1066 Arthropoda and 1658 Insecta single copy gene orthologs, approximately 93% and 91%, respectively, were present in the three draft genomes (Table 1, Table S1). Notably, the assemblies were smaller in size than expected^31^; however, read mappings and BUSCO results signify largely complete and high quality (albeit fragmented) genome assemblies. A complete BUSCO report is detailed in Table S1. The assembled genomes and raw reads have been deposited in GenBank and the SRA (BioProject ID PRJNA575047 and SRP238163, respectively).

**Table 1:** Summary of de novo genome assemblies of the AF, TF and M genomes, read mapping statistics, BUSCO completeness assessment results, number of predicted genes and the percent of repetitive elements detected in each genome.

	Arrhenogenic Female (AF)	Thelygenic Female (TF)	Male (M)
No. of processed reads	134,541,815	102,695,597	85,597,908
N50 (bp)	4101	3889	4164
Mean contig (bp)	2588	2606	2638
No. of contigs	114,048	107,111	109,341
No. of mapped reads (%)	127,991,372 (95.13%)	98,025,293 (95.45%)	82,377,083 (96.24%)
Mean coverage	43X	34X	28X
Estimated genome size (Expected: 426 Mb^31^)	295,268,734 bases 295 Mb	279,238,173 bases 279 Mb	288,503,435 bases 289 Mb
BUSCO—complete*	993 (93.1%)	989 (92.8%)	994 (93.3%)
Repetitive elements %	6.84%	6.61%	6.89%
No. of predicted genes (mean length, bp)	13,910 3345	13,590 3271	13,798 3233
Predicted genes with BLAST hits (%)	93.45%	94.42%	94.22%

* Arthropoda database of 1066 BUSCO groups. For full list, see Table S1.

For *Chrysomya rufifacies*, the expected genome sizes were the same for the two sexes at 425Mbp^31^, yet the assembled genome sizes were 295, 279 and 289 Mbp for arrhenogenic female, thelygenic female, and male, respectively. This amounts to roughly 150 Mbp of ‘missing’ assembled genome. Such discrepancies are not uncommon when sequence- or molecular-based estimates are compared to cytometric estimates of genomes size. The genome of *Arabidopsis thaliana* was originally underestimated to be roughly 115 Mbp^42^ vs. a revised/accepted genome size of 157 Mbp^43^ based on flow cytometry. This is typically attributed to genomes with large proportions of repetitive sequences or unsequenced or unassembled heterochromatic regions. The sequenced-based estimate of the *Drosophila melanogaster*, a relatively small and repeat depauperate genome, have been supported through follow-up work^44^. Results diverge in species with larger genomes though; a previously assembled blow fly genome (*Phormia regina*^38^), assembled close to the expected size (assembled larger at 550/534 Mbp vs. ~ 529/518 Mbp expected for the female and male, respectively). Another example, the assembled genome size of *Lucilia cuprina* was 458 Mbp^45^, smaller than the expected 665/568 Mbp for the female and male, however, an unexpectedly large proportion (57.8%) was attributed to the repetitive landscape of the genome. Generally, the genome sizes of Calliphoridae range from 425 Mbp (*Chrysomya rufifacies*) to 770 Mbp (*Protophormia terranovae*) based on flow cytometry^31^.

**Figure 2:**
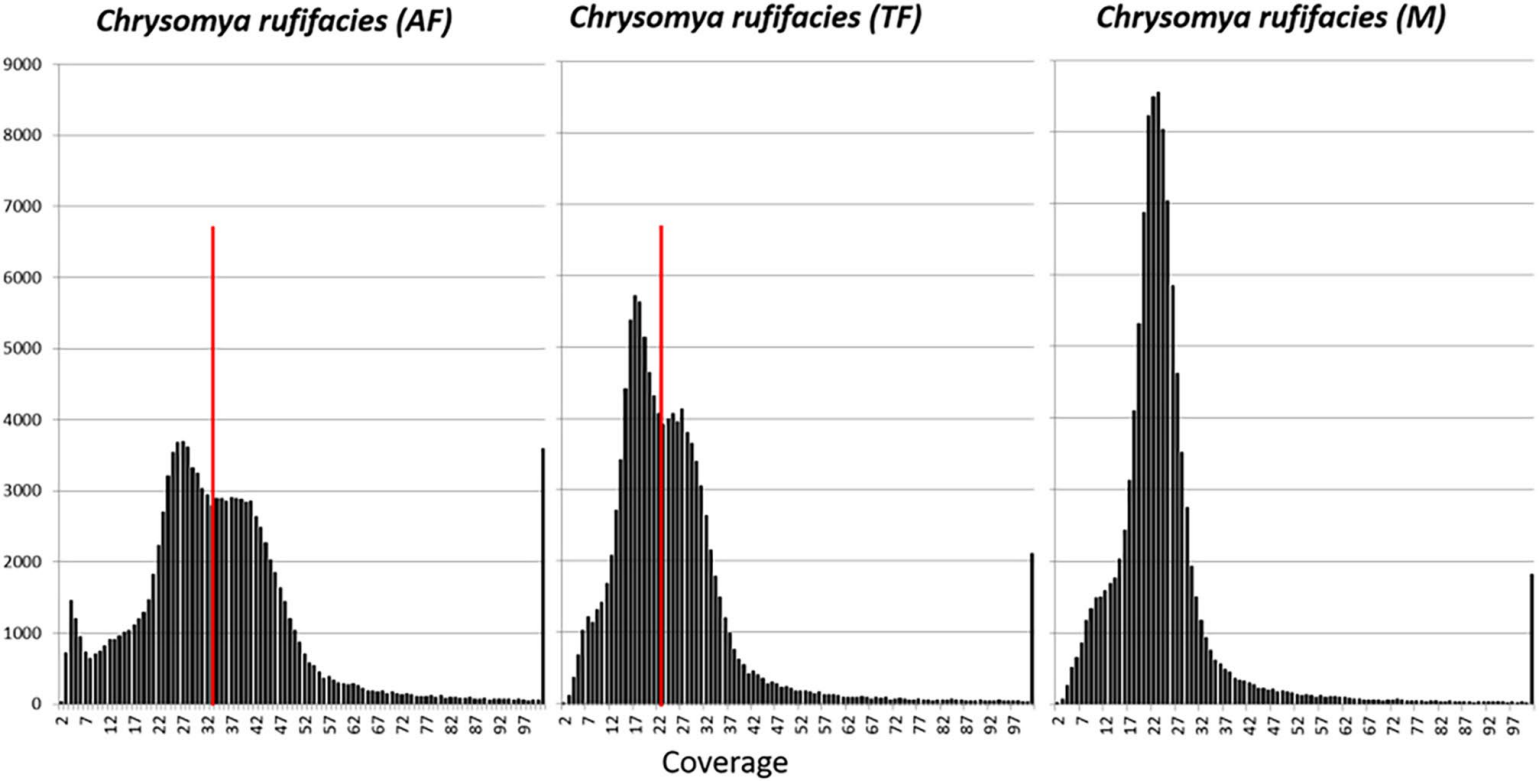
Coverage distributions for the different genomic assemblies with coverage (x-axis) vs. the number of assembled contigs at each coverage. A unimodal distribution is observed in the male genome, while a clear bimodal distribution of the main component of the coverage distribution is observed in females. The different types of females exhibit different ratios of major and minor peak heights.

However, presence of repeat content does not appear to be the case with *C. rufifacies*, as < 7% of the assembled genome is attributed to the repetitive landscape (see results below). Another potential explanation for the discrepancies in genome sizes is the potential for large duplicated chromosomal segments^46,47^. If a/some chromosome(s) has/have duplicated, one would expect to see parts of the genome containing twice as much coverage as the unduplicated portions. We generated frequency distributions of coverage across each genome and visualized this data in Fig. 2 with data represented in Table S2. For both the female genomes, it was obvious there were two distributions of data from the genome, and visually inserting a coverage cutoff, each side of the distribution was analyzed for coverage statistics as well as for the number of variants (Table S2). When considering each side of the distribution, it is apparent that the right skewed distribution (> coverage levels) are roughly 2X the coverage of the left side. Considering duplication theory, if the left side represents 1X and the right 2X, the approximate genome sizes would be 469 Mbp and 434 Mbp for arrhenogenic females and thelygenic females respectively. Another potential explanation for this pattern may be if there is polyploidy or underreplication in the tissues used to produce the genomic sequence data (for a review, see^48^). This study used heads, which are typically considered to lack tissues with these features^49^. It is interesting to note that each sex/type exhibited a different pattern of major to minor peak heights, which may be a clue in deciphering the sex chromosomal dynamics of the species. The results reported here are limited to the largely non-repetitive portions of the genome, though these results suggest a need for further assessment of repetitive regions of the genomes.

### Comparative analysis of predicted genes

**Figure 3:**
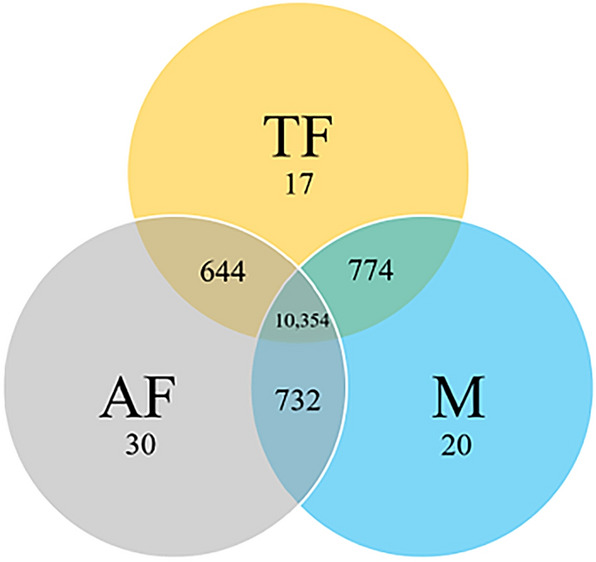
A Venn diagram^50^ displaying the number of orthologous clusters of the predicted protein sequences (i) shared among the three sexes, (ii) shared between any two sexes and (iii) those uniquely found in each group. Cluster classification was done according to sequence analysis data, protein similarity comparisons, and phylogenetic relationships.

Orthologous protein sequence clusters were identified and annotated using OrthoVenn^50^ as seen in Fig. 3 and Additional File 1. A total of 10,354 orthologous clusters were shared among the two females and the male totaling to 15,596 protein sequences shared among the three sexes with average lengths of ~ 425 amino acids/protein. Generally, paired groups shared similar clusters (AF-M: 732 clusters; TF-M: 774 clusters, and AF-TF: 644 clusters), with a small number of unique clusters (TF: 17 clusters, AF: 30 clusters, M: 20 clusters, Fig. 3, Table S3). In all three genomes, the average lengths of the unique protein sequences were ~ 160 amino acids and are therefore are most likely sequencing and assembly artifacts. These unique clusters were analyzed for enriched GO-terms (*p*-value < 0.05; Additional File 2. Unsurprisingly, the shared orthologous protein sequences between the two females show five clusters annotated as yolk protein genes, which is described as the major yolk protein of eggs used as a food source during embryogenesis in *Drosophila*^51^, and typically found on X chromosome in *Drosophila*^52^. Due to its absence in the male genome, it is possible that these genes are part of a region which has differentiated from the “Y” chromosome, or perhaps in a region that did not assemble well, though it is unclear if these are just linked to a causal factor or the causal factor themselves. A comparative analysis on the male and female protein sequences of a different blow fly species—*Phormia regina* showed a similar pattern, with a total of 15,595 proteins sequences shared between the two sexes, and a smaller number, but considerably greater than the *C. rufifiacies* sexes, of unique protein sequences for each sex (*P. regina* female: 727 and *P. regina* male: 1480)^38^. Some of the protein sequences within the unique gene sets are likely to be involved in sex specific developmental trajectories as some functional annotations were found to contain sex specific gene ontology terms for example sperm motility in the unique male gene set, and immune response terms in the female unique set^38^. It is therefore possible that the unique and shared gene sets in *C. rufifacies* could offer clues on the differences within the genomes of the three sex types. A complete list of the orthologous clusters and their putative functional annotations can be found in Additional File 2.

### Sex chromosome genomic characterization

Using read coverage ratios (chromosome quotient, CQ) to compare the male and female genomes and their associated reads, it is possible to isolate genomic regions that are characterized as differentiated, such as would be the case with sex chromosomes^15,53,54^. Based on flow cytometry measurements of genome size differences in male and females (= no difference)^31^, it was not expected that a large portion of the genomes would be isolated using the CQ approach unless the X and Y chromosomes were well differentiated. With 650 and 1590 contigs isolated as putative X and Y chromosomes, respectively, which resulted in ~ 3.3 Mb and ~ 1.5 Mb of genomic differentiation, it appears that (based on sequence data) the genomes contain largely undifferentiated sex chromosomes. Assuming the isolated genomic regions are a part of a differentiated region on putative sex chromosomes, their annotations via BLASTn hits (E-value cutoff ≤ 1E−5) resulted in 86% of the putative X sequences and 29% of the putative Y sequences being annotated.

A significant portion of the sequences with BLASTn results (42.4% in the X chromosome, and 30.8% in the Y chromosome) corresponded to repetitive sequences. This included BAC sequences from *Calliphora vicina* achaete-scute complex, AS-C (Accession Numbers LN877230-LN877235), and microsatellite clone sequences from both *Chrysomya albiceps* (Accession Numbers DQ478598, DQ478605) and *Haematobia irritans* (Accession Number EF629377). In *Ca. vicina,* the *AS-C* gene complex is flanked by repeats and transposable elements^55^. Additionally, within Diptera, the AS-C gene complex (which is made up of the genes *achaete*, *scute*, *lethal of scute*, and *asense*) is located on the X chromosomes in *Drosophila* and is involved in the sex-determining pathway wherein *scute* is an X chromosome signaling element^56^.

The remaining portion of putative X sequences included 16 sequences with hits on yolk protein genes (*L. cuprina* yolk protein D (*ypD*), yolk protein A (*ypA*) and yolk protein B (*ypB*) genes, Accession Number GU109181, and one from *Calliphora erythrocephala* yolk protein 3, Accession Number X7079), two sequences with a hit to the *no bloke* (*nbl*) gene (Accession Number MH173327), nine sequences corresponding to *HSP70* gene (Accession Number HQ609501) and 2 sequences with hits on paired box protein Pax-6-like (*eyeless* in *Drosophila*) gene (Accession Numbers XM_023446990 and XM_023450490) (Additional File 3). Within higher Diptera, yolk protein accumulates in oocytes to be used during embryogenesis and development^52,57^. Genetic and molecular studies in *D. melanogaster* and *L. cuprina* have shown that *yp* genes are specifically expressed in females^52,58,59^ though in *Drosophila* (where there has been more work on the topic), there is evidence of low *yp* expression in males^60–62^ and sperm^63^. Binding sites belonging to the sex-determining gene *doublesex* (*dsx*) have been found on *yp* genes signifying its role in sex specific regulation^52,59,64^. The presence of homologous *yp* sequences in *C. rufifacies* putative chromosome X sequences indicates that these genes are also female specific or female biased in *C. rufifacies* and possibly maintained on a small neo-X region of a chromosome*.* The gene *no bloke* (*nbl)* in *L. cuprina*^65^*,* a homolog of *D. melanogaster’s protein of fourth* (*pof*) gene^66,67^ (an RNA binding protein involved in dosage compensation by targeting the ancestral dipteran sex chromosome (chromosome 4) and chromosome X in *D. melanogaster*) was one of the BLAST hits on 2 putative chromosome Y sequences. In both *L. cuprina* and *D. melanogaster,* this gene has been found to be essential in both male and female viability and fertility^65,67^.

Homologous sequences of *L. cuprina’s* heat shock protein *hsp70* were found in 9 sequences in putative chromosome Y. The promoter region of the *hsp70* gene has been used in sterile insect technique (SIT) studies to develop molecular conditional female lethal genetic modifications^68^. In mammals, *hsp70*-*Sox9* interactions have been implicated in sex determination with a complex formed at sites where SOX9 binds DNA^69^. A member of the family is reported as testis enriched in an eel^70^.

In the putative Y chromosome contigs, 12.3% (57 sequences) of the BLASTn results had hits to the bacteria *Serratia marcescens* (NZ_HG326223, NZ_ALOV00000000, NZ_ATOH00000000)*.* The presence of homologous sequences in *C. rufifacies* to these set of genes from the BLAST results in both the male and female putative sex sequences raises the possibility that a microbial genome may be involved in sex determination and differentiation in *C. rufifacies* as is seen in the isopod *Armadillidium vulgare* (Crustacea, Isopoda), where a chromosomal insertion of a *Wolbachia* genome drives sex determination^71^, though it may also be possible that these are just symbiont sequences that escaped computational filters. Additionally, the signal could be similar to a system observed in *C. elegans*, where lineages that self-fertilize are more sensitive to *S. marcescens* than those that outcross^72^.

### Muller element F is not X-linked in *C. rufifacies*

Chromosomal gene contents, commonly known as Muller elements A to F in *Drosophila*^73^, are thought to be highly conserved across Diptera^15,73^. Muller element F, the dot/fourth chromosome in *Drosophila*, is considered the ancestral X chromosome in many major fly lineages^15,73,74^. Whole genomes of some non-drosophilid insect species which exhibit stable X–Y differentiated sex chromosomes were analyzed and it was determined that genes located on the *Drosophila* dot chromosome are X-linked in these species^15^. In *Drosophila,* however, Muller F reverted back to an autosome more than 60 million years ago but has maintained many characteristics similar to a former X chromosome^74,75^. Muller element F in most Calliphoridae segregates as the sex chromosome, and a dominant male determiner factor located on the Y chromosome directs differential expression of sex determining genes down the male path, leading to distinct structural differences^25,27,76^. In species in which the Muller element is sex-linked, one would expect to observe half as many sequencing reads to map to the reference sequences in males compared to females. When mapping male and female reads (both AF and TF) to each Muller element (A-F), less than 5% of the orthologous contig sequences segregated as X-linked to Muller elements (including Muller element F) (Table S4). Instead an autosomal characteristic sequence coverage distribution is observed in all the Muller elements confirming the high likelihood of undifferentiated sex chromosomes in *C. rufifacies* and introducing a lineage within Calliphoridae in which Muller element F is not the predominant sex-linked element. These results also provide evidence that the sex determination region may be a small region within the genome not easily detectable using coverage differentiation of euchromatic regions of the genome.

### Repetitive landscape

**Figure 4:**
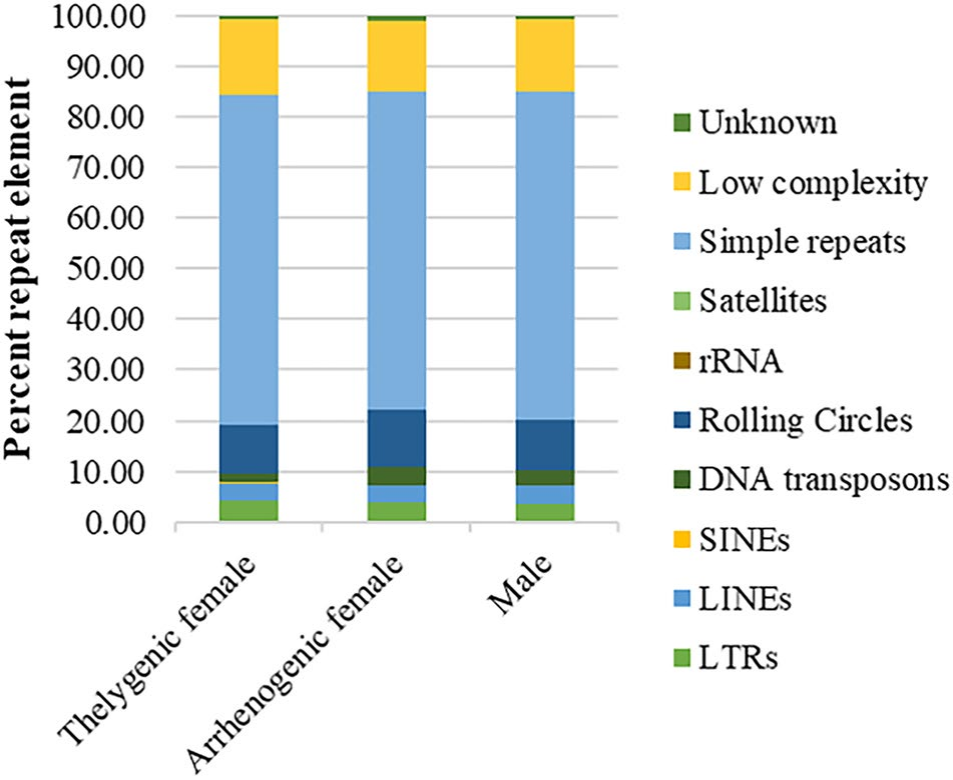
The graph shows the percentage of repeat elements composing the repetitive landscape in each sex type of C. rufifacies. Retrotransposons composed of SINEs, LINEs and LTRs occupied approximately 7% of the total repeatome, while DNA transposons occupied approximately 3% of the repeatome in the male and male producing females and ~ 2% in the female producing females. Satellites and rRNA can barely be seen on the graph as they occupied only 0.07% and 0.05% of the repeatome respectively. Simple repeats were the predominant repetitive element occupying almost 65% of the whole repetitive landscape.

Repeat sequences have recently been found to be important precursors and contributors to eukaryotic genome’s architecture, stability, evolution and environmental adaptation^77,78^. In *Stomoxys calcitrans*, the Muller element suspected as the sex chromosome seems to exhibit a distinct repeat element pattern^79^. The amount of repetitive DNA among insect species varies greatly^38,80–82^. Some insects have greater than 50% of their genome occupied by repetitive elements (American cockroach, *Periplaneta americana*^82^) while others have less than 10% (*Phormia regina,* black blow fly^38^). The assembled portion of the *C. rufifiacies* genome has a small proportion of repetitive elements in the assembly, accounting for 6.61% (18 Mb), 6.84% (20 Mb) and 6.89% (19 Mb) of the TF, AF and M assembled genomes, respectively (Table 1, Table S5). The predominant repetitive elements were simple repeats, which occupy approximately 4.3% (~ 12.5 Mb) of the *C. rufifacies* genomes. The remainder of the repetitive landscape comprised of ~ 0.5% of DNA retrotransposons (LTRs, LINEs and SINEs), ~ 0.2% DNA transposons (hAT, CMC, Maverick, Kolobok, Mule, P, PIF, PiggyBac, Sola, TcMar, Zator), ~ 0.7 rolling circle, ~ 1% low complexity regions and ~ 0.06% unknown repetitive sequences (Fig. 4). In the characterized putative sex chromosomes, 6.17% of the X chromosome (~ 204 kb) and 2.77% of the Y chromosome (41.90 kb) were repetitive elements.

## Conclusion

Rapid diversification caused by changes in evolutionary processes has introduced variation in sex-determining mechanisms between and within species^15,83,84^. The family Calliphoridae is an excellent model for evaluation of sex chromosome evolution as both homomorphic (*C. rufifacies, C. albiceps*^33,35^), and heteromorphic (*L. cuprina, P. regina*^32^) sex chromosomes are observed among closely related species. Additionally, while a majority of blow flies are amphogenic (females produce an equal ratio of male and female progeny), others, such as *C. rufifacies* possess a distinct monogenic (females produce unisexual progeny^35,39^) system, with two type of females (arrhenogenic and thelygenic^35,39^) and the sex of the offspring is determined by the maternal genotype^39^. This may be in response to selective pressures with respect to inbreeding – producing unisexual offspring guarantees full siblings will not mate with each other, thus resulting in a genetically robust population even when population numbers begin to decline. Gall midges^85^, Hessian flies^86^, and certain populations of *Musca domestica*^87^ have monogenic life histories, all of which is likely related to controlling for inbreeding depression, not uncommon when resources are scarce and unpredictable. Therefore, the presence of the individual sex draft genomes herein will facilitate addressing questions on the origination and evolution of the diversity of sex-determining mechanisms observed within Calliphoridae.

As calliphorids are decomposers and filth flies^88^, many of this group’s adaptations have also resulted in their classification as agricultural pests^89^ and for their utility in forensic entomology investigations^90^. The function of many Calliphoridae as decomposers of animal remains also means they are important nutrient recyclers^91,92^, which are becoming of greater interest in decomposition ecology as most research has been focused on autotrophic biomass^93^. Therefore, the addition of these draft genomes and the predicted protein-encoding genes will expand the taxonomic breadth of study organisms and provide unique insights into the molecular biology, ecology, and evolution of blow flies. This, in cooperation with genomic evaluations of other dipteran species, will contribute in the exploration and provision of new targets for pest control strategies based on controlling specific sexes. Currently, the sterile insect technique is still in use to control the primary screwworm fly (*Co. hominivorax*) in which males are irradiated and released into the environment^94^. However, these mass production facilities must rear male and female offspring due to the reproductive biology of this species and difficulty differentiating between the sexes in the immature stages, resulting in production of a sex that is not even used and is thus discarded. Understanding the mechanism in which a single sex is produced, and being able to genetically modify other calliphorid species to include this switch, could provide both economic and agricultural benefits^95,96^.

In conclusion, this new genome consisting of three draft genomes of two females types and males represent additional genomic resources of a calliphorid fly with economic, agricultural, forensic and medical importance. The genomes identify an important link in the study of evolution and diversification of sex-determining systems. We provide evidence for a loss of sex chromosomes, or the movement of very small components of ancestral sex chromosomes to autosomes, as there is little evidence for sex chromosomes in the genome (though some contigs identified do align with traditional sex-determining chromosomes) and no obvious pattern in Muller element allocation of such sites. Several interesting hypotheses regarding the sex-determination mechanism of this species arise from this work including the role of the *no blokes* / *painted on the fourth*, *scute*, *yolk proteins*, and potentially inserted *Serratia marcescens* genes in this unique monogenic sex-determination system with seemingly no (or very small and possibly neo) sex chromosomes. Interestingly, canonical sex determination genes (*transformer* and *Musca domestica male determiner*) either produced truncated proteins when annotated (*tra*) or did not align (*Mdmd*) with our genomic scan for sex-determining elements. These results are similar to a previous chromosomal staining experiments in the species that only found evidence for *daughterless* near a suspected sex-determining translocation^97^, though it is worth noting that a full accounting of *Drosophila* sex determination loci was lacking at the time of that experiment. It is also worth noting that *daughterless* and *scute* (identified as a putative X chromosomal sequence here) interact in *Drosophila*^98^, providing (along with the *no blokes* location on a putative Y chromosomal contig) some evidence that a dosage compensation-like molecular function^99^ may be important in *C. rufifacies* sex determination. This hypothesized role of dosage compensation coincides with the observed differences in genomic coverage between males and females, where females exhibit two peaks in coverage and males exhibit one. Furthermore, *eyeless* (*Pax-6*) is known to interact with both *daughterless*^100^ and there is some support for it interacting with *doublesex*^101^ in *Drosophila*; deepening the original support for a role of *daughterless* in the *Ch. rufifiacies* sex determination system. Additional connections of identified targets include *hsp70*-*Sox9* regulation of sex in some systems^69^ and common co-regulation by Pax/Sox genes in a variety of systems^102^. Additional work on the functional annotation of the sex-determining cascade of genes, as well as the identification of the master switch in *Ch. rufifacies*, will lead to invaluable and potentially wide-ranging implications across evolutionary biology. Although these genomes have some limitations (mostly fragmented genomes), the genomes and identified targets here are ideal starting points for more detailed dissections of this sex determination mechanism and sex chromosome evolution.

## Methods

### DNA library preparation and sequencing

Pooled genomic DNA was extracted from the heads of five male-producing females (arrhenogenic), five female-producing females (thelygenic) and five male flies originating from a lab colony of *Chrysomya rufifacies* (see^37^ for colony foundation, maintenance, and sample collection procedures) using the DNeasy Blood and Tissue DNA Extraction kit following the manufacturer’s instructions (Qiagen Inc., Valencia, CA, USA). Each extract was quantified using a Qubit fluorimeter (Thermo Fisher Scientific, Waltham, MA, USA) so that a total of 1 µg of genomic DNA was sent to a facility for library preparation. Libraries (N = 3) were constructed following the TruSeq DNA Sample Preparation Guide by Illumina (Catalog #PE-940-2001. Part # 15,005,180 Rev. A, November 2010). Sequencing was performed on the three paired-end libraries using the Illumina HiSeq2000 sequencing platform (Illumina Inc, San Diego, CA, USA) with a read length of 2 × 100 bp. Both of the library preparation and sequencing was completed by the Purdue University Genomics Core Facility (West Lafayette, IN, USA). The three libraries were multiplexed on a single lane. All sequencing data produced in this study have been deposited in the National Center for Biotechnology Information Sequence Read Archive (NCBI SRA) and can be accessed under the BioProject ID PRJNA575047 and SRA Accession Number SRP238163.

### Pre-processing and quality trimming

Raw reads were trimmed to eliminate low quality reads (Phred score < 20) and adapter sequences. On a per-library basis, overlapping pairs of reads were merged into a single sequence read creating longer and higher quality reads. Mismatch cost was set to 2, gap cost was set to 3, and the minimum score required for an alignment to be accepted for merging was set to 8. Both read trimming and merging were analyzed using the software CLC Genomics Workbench (CLC-GWB v9) (Qiagen Inc.). Extraneous or contaminating DNA were filtered out by mapping the merged and trimmed reads to 3,006 phage (www.phantome.org, v2016-04-01) and 49,290 bacterial genomes (www.ncbi.nlm.nih.org, downloaded on 05/2016 and 03/2017). Mitochondrial reads were subsequently removed by mapping the reads on to the mitochondrial genome of *C. rufifacies* (NC_019634.1). The resulting unmapped reads were thereafter used in the de novo assembly step.

### Genome assemblies, scaffolding and evaluation

De novo genome assemblies were performed on each of the three processed and quality filtered libraries (male, arrhenogenic female and thelygenic female) using the CLC-GWB v9 assembler. Several iterations of the de novo assemblies were carried out with k-mer sizes ranging between 24 – 50 nucleotide, and bubble sizes ranging from 100–1000; with the intention of selecting the ideal assembly with optimal parameters to be used in downstream analysis. Optimal k-mer sizes for all three sets of libraries was determined to be 32 bp. Additionally a transcriptome of the thelygenic female was also assembled for scaffolding purposes only, using a k-mer size of 32 bp. For all the assemblies, a mismatch cost of 2, insertion cost of 3 and deletion cost of 3 was selected. Mapping parameters were set such that 50% of each read needed to have at least 90% identity to be included in the final mapping. Contigs from each of the three assembled draft genomes were scaffolded with the assistance of the assembled thelygenic transcriptome using the scaffolding program LRNA scaffolder^103^. This program uses transcriptome contigs to orient and combine genomic fragments. Calculations of the assembly statistics was done by CLC-GWB v9 and the genome assessment tool QUAST v3.1^104^. Coverage mapping and subsequent variant detection was done by mapping reads to the assembled genomes ignoring positions with coverage > 100,000, and ignore broken paired reads. Data were visualized using Microsoft Excel using frequency distributions. Universal single copy orthologs (USCOs) was used to assess completeness and contiguity of the assembled genomes using the Benchmarking Universal Single-Copy Orthologs (BUSCO) v2.0.1^105^. BUSCO measures the fraction of genes highly conserved in related species by mapping and identifying them using a database of orthologs (OrthoDB) from eukaryotes, diptera, arthropods and insects.

### Gene prediction, annotation and ontology

Ab initio prediction of gene and protein sequences for each of the three sex types was performed by the gene predicting progam Maker^106^ on the three draft genomes. The flag option ‘always_complete’ in the maker_opts.ctl file was set to 1, the rest of the parameter were left at default settings. To infer gene predictions, expressed sequence tag (EST) evidence for gene transcription was obtained from the assembled thelygenic transcriptome and alternate EST evidence from *D. melanogaster* gene sequences (GCF_000001215.4_Release_6_plus_ISO1_MT_rna). Additional evidence was obtained from protein sequences of *L. cuprina* (GCA_001187945.1_ASM118794v1_protein)*, D. melanogaster* (GCF_000001215.4_Release_6_plus_ISO1_MT_protein) and arrhenogenic female protein sequence (from a previous gene prediction run not published). Gene sequences which encoded peptide sequences ≥ 30 amino acids in length were filtered and preserved. RNA-seq reads from the thelygenic female (Accession Number SRX149675) were mapped onto the gene sequences predicted for each of the three sex types following the same mapping parameters used in the genome assembly process. Annotation was performed using a non-redundant arthropoda protein BLAST database (BLASTp v2.2.28+) with an E-value cutoff of ≤ 1E-5^76^. The web platform OrthoVenn^50^ was used to identify overlap among orthologous clusters from the predicted protein sequences of the two females and the males in a genome wide perspective. The predicted protein sequences for the thelygenic female, arrhenogenic female and the male were uploaded onto OrthoVenn independently in fasta format and default parameters were used to run the analysis. Orthologous clusters that were unique to each sex type, shared between the two females, shared between each of the females and the male, and common in all three were grouped together. The cluster classification was done according to sequence analysis data, protein similarity comparisons, and phylogenetic relationships^50^. OrthoVenn deduced the putative function of each orthologous cluster by performing a protein BLAST search against a non-redundant protein database in UniProt. Top hits with an e-value of < 1E-5 were defined as the putative function of each cluster^50^.

### Sex chromosome characterization

Putative X and Y chromosome sequences were characterized using the chromosome quotient approach^53^ which utilizes read coverage ratios of alignment to differentiate X, Y and autosomal sequences. The chromosome quotient program^53^ was used to align male and female reads onto each other’s genome (male reads independently mapped to male genome and to each of the female genomes, and vice versa ). A stringent aligning criterion requiring a whole read to map onto the reference contigs with zero mismatch was done in order to reduce the number of false positives that may be caused by the highly repetitive sequences from Y chromosomes with closely related sequences on the autosomal or X chromosomes due to duplication events^53,54^. Chromosome quotients were calculated by comparing the number of alignments from female sequence data to male sequence data. Ideally, putative X sequences were expected to have a CQ ratio of 2 with X sequences characterized as those with twice as many female reads aligned as male, while putative Y sequences a CQ ratio of 0. Due to the presence of the two types of females (thelygenic and arrhenogenic), the CQ approach was implemented on each female independently resulting to two sets of X and Y sequences. Male contig sequences with a CQ of less than 0.3X were grouped as putative Y chromosomes to accommodate repetitive Y sequences that may be present in both the male and female. A total of 2,195 contigs (~ 2 Mb from male and arrhenogenic female comparison) and 4,031 contigs (~ 4 Mb from male and thelygenic female comparison) were identified as putative Y chromosomal sequences. The two predicted sets of putative Y sequences were compared to determine the proportion of overlap shared between them. Female contig sequences with a CQ ranging between 1.6X and 2.5X were grouped as putative X sequences. This CQ interval was selected to reduce false positives. A total of 23,624 contigs (~ 64 Mb) and 7448 contigs (~ 15 Mb) from the arrhenogenic and thelygenic female respectively, were categorized as putative X chromosomes. A comparative analysis of both sets of putative X chromosomes was performed by CD-HIT-2D-EST v4.5.6^107,108^, to isolate a representative set of *C. rufifacies* chromosome X sequences characterized by both females, using a length difference cutoff and a sequence identity cutoff both of 80%. A nucleotide BLAST (BLASTn v2.6.0+, E-value cutoff ≤ 1E−5) was performed on the characterized sex chromosome sequences using a non-redundant nucleotide database^109^. Resulting BLAST results were functionally characterized using default parameters on Blast2GO v5.1.13^110^ and gene ontology (GO) terms assigned to the BLAST results. The functional categories were simplified using the GO slim functionality in Blast2GO and enrichment analysis using Fisher’s exact test performed on them. The enriched GO terms and their corresponding FDR values were summarized and categorized to the three GO domains: biological processes, cellular component and molecular functions; and visualized using default settings of the REViGO web server^111^.

### X-linked Muller elements

Coding sequences of the chromosomal gene contents (Muller elements A-F) from *Drosophila melanogaster* were downloaded from GenBank. The longest isoforms were selected for each gene resulting to a total of 10,488 coding sequences. They were thereafter queried against the assembled genomes of the male and the two females using a translated nucleotide and database (tBLASTx v2.6.0 + , E-value cutoff ≤ 1E−5) to identify orthologous contig sequences within the genomes. Orthologous contig sequences were assigned as belonging to the respective Muller elements they segregated with. To determine which Muller elements were X-linked in *C. rufifacies,* male and female sequence reads were aligned to the identified orthologous contig sequences using the CLC-GWB v9 read mapper, and the read coverages compared. To reduce false positives, stringent mapping parameters were used such that 100% of each read needed to have at least 80% identity to be included in the final mapping. The program DESeq^112^ was used to identify any differential read coverages observed within the orthologous Muller elements to identify sequences with a twofold higher abundance in females than males, by calculating a Log2(M/F) coverage ratio. Contig sequences with a Log2 (M/F) coverage ratio within the range of -0.6 and −1.3 were considered to be X-linked.

### Repeat sequence analysis

A library of all known Diptera repetitive elements was used to identify repetitive elements in each of the 3 genomes and the putatively characterized X and Y chromosomes using the program RepeatMasker v4.0.7 in default mode.

## Supplementary information

Supplementary file1

Supplementary file2

Supplementary file3

Supplementary file4

Supplementary file5
