# Supplementary material for: The genomes of a monogenic fly: views of primitive sex chromosomes
Source: Sci Rep. 2020 Sep 25;10:15728. doi: 10.1038/s41598-020-72880-0 (PMC7519133; doi:10.1038/s41598-020-72880-0)
Supplement: Supplementary file 4 — Supplementary file4 [file 41598_2020_72880_MOESM4_ESM.docx]

**SUPPLEMENTARY TABLES**

**The genomes of a monogenic fly: Views of primitive sex chromosomes.**

Anne A. Andere^1^, Meaghan L. Pimsler^2^, Aaron M. Tarone^3^, Christine J. Picard^1*^

^1^ Department of Biology, Indiana University- Purdue University Indianapolis, Indianapolis, Indiana, aaandere@iupui.edu

^2^Department of Biological Sciences, The University of Alabama, Tuscaloosa, Alabama, mlpimsler@gmail.com

^3^ Entomology Department, Texas A&M University, College Station, Texas, tamlucilia@tamu.edu

*Corresponding Author: cpicard@iupui.edu

Table S1. BUSCO Completeness report of the male, thelygenic female and arrhenogenic females of *C. rufifacies*. Complete and single-copy means the gene ortholog has been found once as expected and is within 2 standard-deviations in length of the BUSCO meeting the score criteria for the alignment. Complete and duplicated means there's more than one copy found that meets length and score criteria. Fragmented means part of the gene was found but not the full length, and missing means the BUSCO group was not found.

| Thelygenic Female | Eukaryota | Diptera | Arthropoda | Insecta |
| --- | --- | --- | --- | --- |
| Total BUSCO groups | 303 | 2799 | 1066 | 1658 |
| Complete BUSCOs (C) | 278 (91.8%) | 2385 (85.2%) | 989 (92.8%) | 1508 (91.0%) |
| Complete and single-copy BUSCOs (S) | 276 (91.1%) | 2362 (84.4%) | 977 (91.7%) | 1485 (89.6%) |
| Complete and duplicated BUSCOs (D) | 2 (0.7%) | 23 (0.8%) | 12 (1.1%) | 23 (1.4%) |
| Fragmented BUSCOs (F) | 6 (2.0%) | 130 (4.6%) | 15 (1.4%) | 31 (1.9%) |
| Missing BUSCOs (M) | 19 (6.2%) | 284 (10.2%) | 62 (5.8%) | 119 (7.1%) |
|  |  |  |  |  |
| Male | **Eukaryota** | **Diptera** | **Arthropoda** | **Insecta** |
| Total BUSCO groups | 303 | 2799 | 1066 | 1658 |
| Complete BUSCOs (C) | 276 (91.1%) | 2400 (85.8%) | 994 (93.3%) | 1518 (91.5%) |
| Complete and single-copy BUSCOs (S) | 273 (90.1%) | 2384 (85.2%) | 986 (92.5%) | 1504 (90.7%) |
| Complete and duplicated BUSCOs (D) | 3 (1.0%) | 16 (0.6%) | 8 (0.8%) | 14 (0.8%) |
| Fragmented BUSCOs (F) | 7 (2.3%) | 145 (5.2%) | 12 (1.1%) | 24 (1.4%) |
| Missing BUSCOs (M) | 20 (6.6%) | 254 (9.0%) | 60 (5.6%) | 116 (7.1%) |
|  |  |  |  |  |
| Arrhenogenic Female | **Eukaryota** | **Diptera** | **Arthropoda** | **Insecta** |
| Total BUSCO groups | 303 | 2799 | 1066 | 1658 |
| Complete BUSCOs (C) | 272 (89.8%) | 2381 (85.0%) | 993 (93.1%) | 1508 (90.9%) |
| Complete and single-copy BUSCOs (S) | 270 (89.1%) | 2366 (84.5%) | 981 (92.0%) | 1494 (90.1%) |
| Complete and duplicated BUSCOs (D) | 2 (0.7%) | 15 (0.5%) | 12 (1.1%) | 14 (0.8%) |
| Fragmented BUSCOs (F) | 6 (2.0%) | 138 (4.9%) | 17 (1.6%) | 40 (2.4%) |
| Missing BUSCOs (M) | 25 (8.2%) | 280 (10.1%) | 56 (5.3%) | 110 (6.7%) |

Table S2. Data associated with read mapping statistics for the distributions in Figure 2.

|  | *Cruf* AF | | *Cruf* TF | | *Cruf* M |
| --- | --- | --- | --- | --- | --- |
| Mean Coverage (SE) | 52.50 (2.14) | | 36.86 (1.81) | | 34.79 (1.28) |
| Median Coverage | 32.07 | | 21.80 | | 22.17 |
| Total # variants | 2,826,180 | | 2,924,285 | | 2,198,938 |
| Red line delineation | <32X | >32X | <23X | >23X | n/a |
| # contigs | 54,772 | 55,176 | 60,304 | 50,965 | 109,341 |
| Length of contigs (Mbp) | 99 | 185 | 108 | 163 | 289 |
| # reads mapped | 25,506,380 | 129,383,978 | 19,531,466 | 78,785,565 | 85,597,908 |
| # variants | 1,194,952 | 2,631,228 | 838,346 | 3,085,939 | 2,198,938 |
| # variants/1000bp | 12.0 | 14.0 | 7.7 | 12.7 | 7.8 |
| % SNV | 81.0 | 78.6 | 81.7 | 78.9 | 79.6 |
| % MNV | 3.8 | 3.6 | 3.4 | 3.5 | 3.6 |
| % Ins | 6.7 | 8.0 | 6.3 | 7.8 | 7.4 |
| % Del | 7.8 | 8.9 | 7.9 | 9.0 | 8.6 |
| % Replacement | 0.8 | 0.9 | 0.6 | 0.8 | 0.8 |

Table S3. Orthologous clusters of predicted protein sequences from the thelygenic female, arrhenogenic female and the male assemblies. The table shows the number of clusters and the total number of protein sequences within each cluster which is (i) unique to each group, (ii) shared between each pair, and (iii) shared among the three groups. Cluster classification was done according to sequence analysis data, protein similarity comparisons, and phylogenetic relationships (AF = arrhenogenic female, TF = thelygenic female, M = male).

|  | **Unique Clusters** | **Total #protein sequences composing the cluster** |
| --- | --- | --- |
| **Thelygenic Female** | 17 | 35 |
| **Arrhenogenic Female** | 30 | 66 |
| **Male** | 20 | 44 |
| **AF – TF** | 644 | 1,313 (659 TF, 654 AF) |
| **AF – M** | 732 | 1,490 (745 AF, 745 M) |
| **TF – M** | 774 | 1,567 (783 M, 784 TF) |
| **AF – TF – M** | 10,354 | 31,812 ( 10,602 AF, 10,630 TF,10,580 M) |

**Table S4.** Orthologous contig sequences in *C. rufifacies* of the chromosomal gene contents (Muller elements) from *D. melanogaster’s* Muller elements. Male vs female read coverage ratios was used to determine which set of orthologous Muller elements had a 2X sequence coverage (X-linked). Due to the presence of two types of female, read coverage ratios on the male was performed twice (AF-M and TF_M).

|  | **AF** | | **TF** | | **AF-M** | | **TF-M** | |
| --- | --- | --- | --- | --- | --- | --- | --- | --- |
| **Muller elements (*D. melanogaster* chromosome)** | No. of contigs with tBLASTx hits | No. of X-linked contigs (%) | No. of contigs with tBLASTx hits | No. of X-linked contigs | No. of contigs with tBLASTx hits | No. of X-linked contigs | No. of contigs with tBLASTx hits | No. of X-linked contigs |
| **A (Chr X)** | 1,454 | 3  (0.21%) | 1,454 | 26  (1.79%) | 1,464 | 3  (0.20%) | 1,464 | 16  (1.09%) |
| **B (Chr 2L)** | 1,720 | 9  (0.62%) | 1,737 | 28  (1.61%) | 1,706 | 6  (0.35%) | 1,706 | 32  (1.88%) |
| **C (Chr 2R)** | 1,917 | 12  (0.63%) | 1,913 | 26  (2.40%) | 1,896 | 8  (0.42%) | 1,896 | 29  (1.53%) |
| **D (Chr 3L)** | 1,782 | 1  (0.10%) | 1,835 | 45  (2.45%) | 1,831 | 10  (0.55%) | 1,831 | 31  (1.69%) |
| **E (Chr 3R)** | 2,128 | 11  (0.51%) | 2,131 | 51  (2.39%) | 2,100 | 7  (0.33%) | 2,100 | 32  (1.52%) |
| **F (Chr 4)** | 77 | 1  (1.30%) | 85 | 3  (3.53%) | 84 | 0  (0%) | 84 | 1  (1.19%) |

**Table.S5.** A summary of the percent of the genome composed of repetitive sequences in the thelygenic female, arrhenogenic female and the male *Chrysomya rufifacies,* their copy number and the number of bases of each. Repetitive elements were identified using homology to known Diptera repetitive elements

|  |  | **Thelygenic Female** | | | **Arrhenogenic Female** | | | **Male** | | |
| --- | --- | --- | --- | --- | --- | --- | --- | --- | --- | --- |
|  |  | **Copy number** | **Bases (bp)** | **% Genome** | **Copy number** | **Bases (bp)** | **% Genome** | **Copy number** | **Bases (bp)** | **% Genome** |
| **Class 1 Retrotransposons** | | | | | | | | | | |
| LTRs | Copia | 441 | 104,256 | 0.04 | 463 | 106,151 | 0.04 | 456 | 103,567 | 0.04 |
|  | Gypsy | 3,958 | 471,065 | 0.17 | 3,816 | 463,860 | 0.16 | 3,532 | 440,356 | 0.15 |
|  | Pao | 1,130 | 176,457 | 0.06 | 1,131 | 187,231 | 0.06 | 1,119 | 186,236 | 0.07 |
| LINEs | CR1 | 254 | 22,554 | 0.00 | 264 | 23,764 | 0.01 | 260 | 23,089 | 0.01 |
|  | Dong-R4 | 10 | 706 | 0.00 | 10 | 721 | 0.00 | 8 | 553 | 0.00 |
|  | I | 2,743 | 319,008 | 0.11 | 2,875 | 337,734 | 0.11 | 2,752 | 318,547 | 0.11 |
|  | L1 | 84 | 4,898 | 0.00 | 11 | 5,971 | 0.00 | 88 | 4,900 | 0.00 |
|  | L2 | 309 | 26,244 | 0.01 | 318 | 27,745 | 0.01 | 301 | 26,002 | 0.01 |
|  | Penelope | 4 | 260 | 0.00 | 7 | 356 | 0.00 | 3 | 148 | 0.00 |
|  | R1 | 1,273 | 190,621 | 0.07 | 1,350 | 202,744 | 0.07 | 1,343 | 198,440 | 0.07 |
|  | R2 | 8 | 1,847 | 0.00 | 8 | 1,869 | 0.00 | 8 | 1,924 | 0.00 |
|  | RTE | 829 | 105,303 | 0.04 | 950 | 121,165 | 0.04 | 887 | 109,982 | 0.04 |
| SINEs | tRNA | 97 | 6,646 | 0.00 | 111 | 7,381 | 0.00 | 179 | 11,402 | 0.00 |
| **Class II DNA Transposons** | | | | | | | | | | |
| Cut and Paste Transposons | DNA | 213 | 99,351 | 0.04 | 265 | 123,658 | 0.04 | 237 | 108,259 | 0.04 |
|  | CMC | 911 | 58,029 | 0.02 | 1,020 | 65,497 | 0.02 | 976 | 61,004 | 0.02 |
|  | hAT | 2,767 | 2,771 | 0.00 | 3,387 | 322,255 | 0.11 | 3,059 | 287,617 | 0.10 |
|  | Kolobok | 5 | 258 | 0.00 | 5 | 255 | 0.00 | 8 | 463 | 0.00 |
|  | Maverick | 173 | 48,891 | 0.02 | 180 | 47,465 | 0.02 | 191 | 53,806 | 0.02 |
|  | Merlin | 0 | 0 | 0 | 1 | 59 | 0.00 | 0 | 0 | 0.00 |
|  | MULE | 67 | 3,842 | 0.00 | 62 | 3,560 | 0.00 | 66 | 3,979 | 0.00 |
|  | P | 245 | 13,844 | 0.01 | 226 | 12,963 | 0.00 | 236 | 13,848 | 0.01 |
|  | PIF | 137 | 6,431 | 0.00 | 164 | 7,692 | 0.00 | 140 | 6,661 | 0.00 |
|  | PiggyBac | 21 | 4,161 | 0.00 | 24 | 4,588 | 0.00 | 23 | 3,970 | 0.00 |
|  | Sola | 30 | 1,629 | 0.00 | 35 | 2,078 | 0.00 | 38 | 2,114 | 0.00 |
|  | TcMar | 607 | 92,035 | 0.03 | 662 | 101,190 | 0.03 | 586 | 92,098 | 0.03 |
|  | Zator | 5 | 364 | 0.00 | 5 | 353 | 0.00 | 6 | 416 | 0.00 |
| Rolling Circle | Helitron | 17,100 | 1,790,559 | 0.64 | 21,232 | 2,269,371 | 0.77 | 18,757 | 1,955,084 | 0.68 |
| rRNA |  | 61 | 9,567 | 0.00 | 74 | 10,276 | 0.00 | 64 | 11,651 | 0.00 |
| Satellites |  | 46 | 11,112 | 0.00 | 58 | 15,714 | 0.01 | 58 | 14,388 | 0.01 |
| Simple repeats |  | 234,230 | 11,974,585 | 4.29 | 253,970 | 12,687,148 | 4.3 | 268,134 | 12,844,756 | 4.45 |
| Low complexity | | 53,832 | 2,766,628 | 0.99 | 55,834 | 2,843,978 | 0.96 | 55,983 | 2,821,282 | 0.98 |
| Unknown |  | 1,788 | 151,518 | 0.05 | 2,131 | 186,861 | 0.06 | 1,987 | 166,619 | 0.06 |
| **Total Repeat Content** | | **323,378** | **18,465,440** | **6.61** | **350,649** | **20,191,653** | **6.84** | **361,485** | **19,873,161** | **6.89** |
