# Supplementary material for: The genomes of a monogenic fly: views of primitive sex chromosomes
Source: Sci Rep. 2020 Sep 25;10:15728. doi: 10.1038/s41598-020-72880-0 (PMC7519133; doi:10.1038/s41598-020-72880-0)
Supplement: Supplementary file 5 — Supplementary file5 [file 41598_2020_72880_MOESM5_ESM.pptx]

## Slide 1
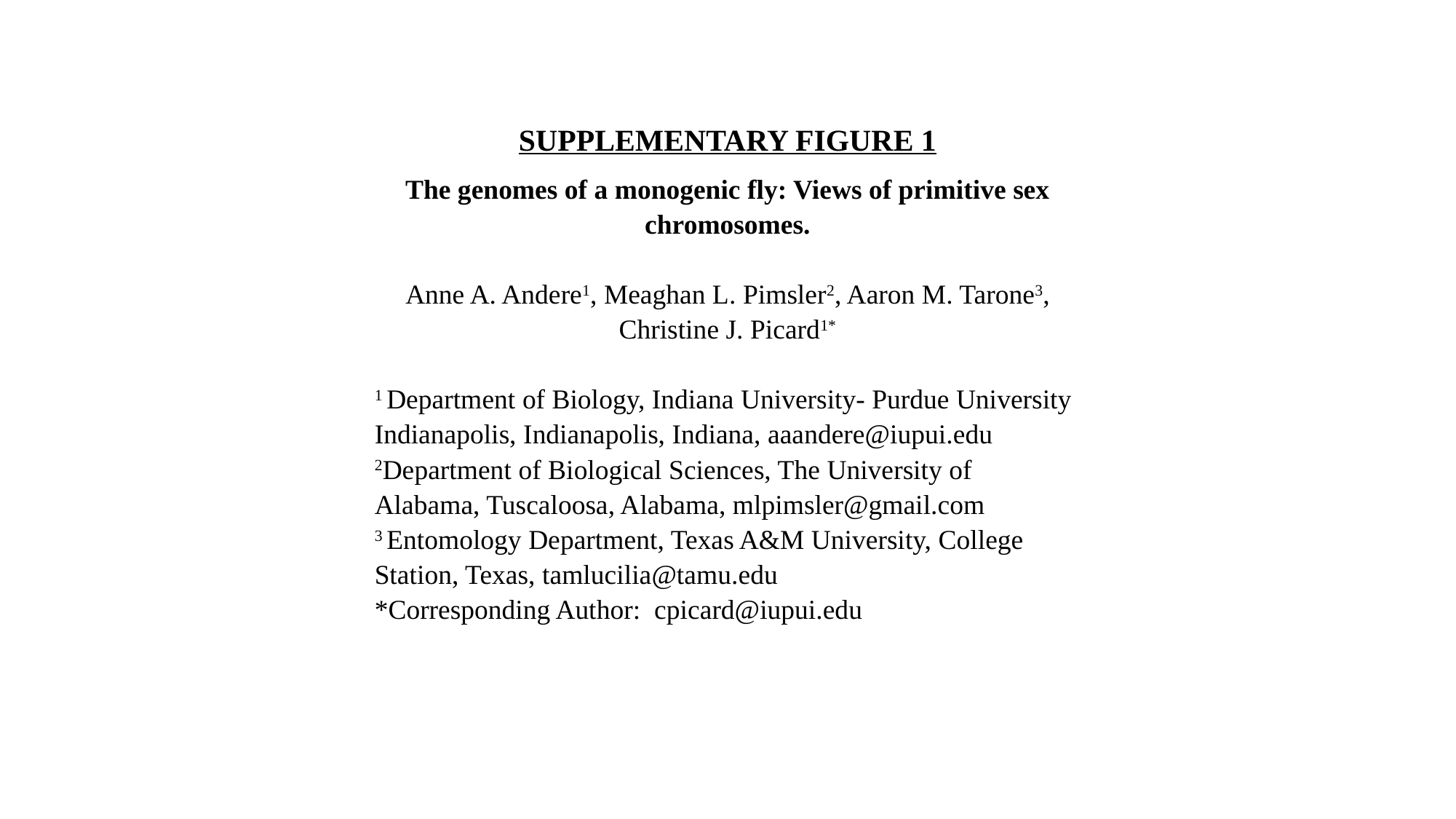

SUPPLEMENTARY FIGURE 1
The genomes of a monogenic fly: Views of primitive sex chromosomes.
Anne A. Andere1, Meaghan L. Pimsler2, Aaron M. Tarone3, Christine J. Picard1*
1 Department of Biology, Indiana University- Purdue University Indianapolis, Indianapolis, Indiana, aaandere@iupui.edu
2Department of Biological Sciences, The University of Alabama, Tuscaloosa, Alabama, mlpimsler@gmail.com
3 Entomology Department, Texas A&M University, College Station, Texas, tamlucilia@tamu.edu
*Corresponding Author: cpicard@iupui.edu

## Slide 2
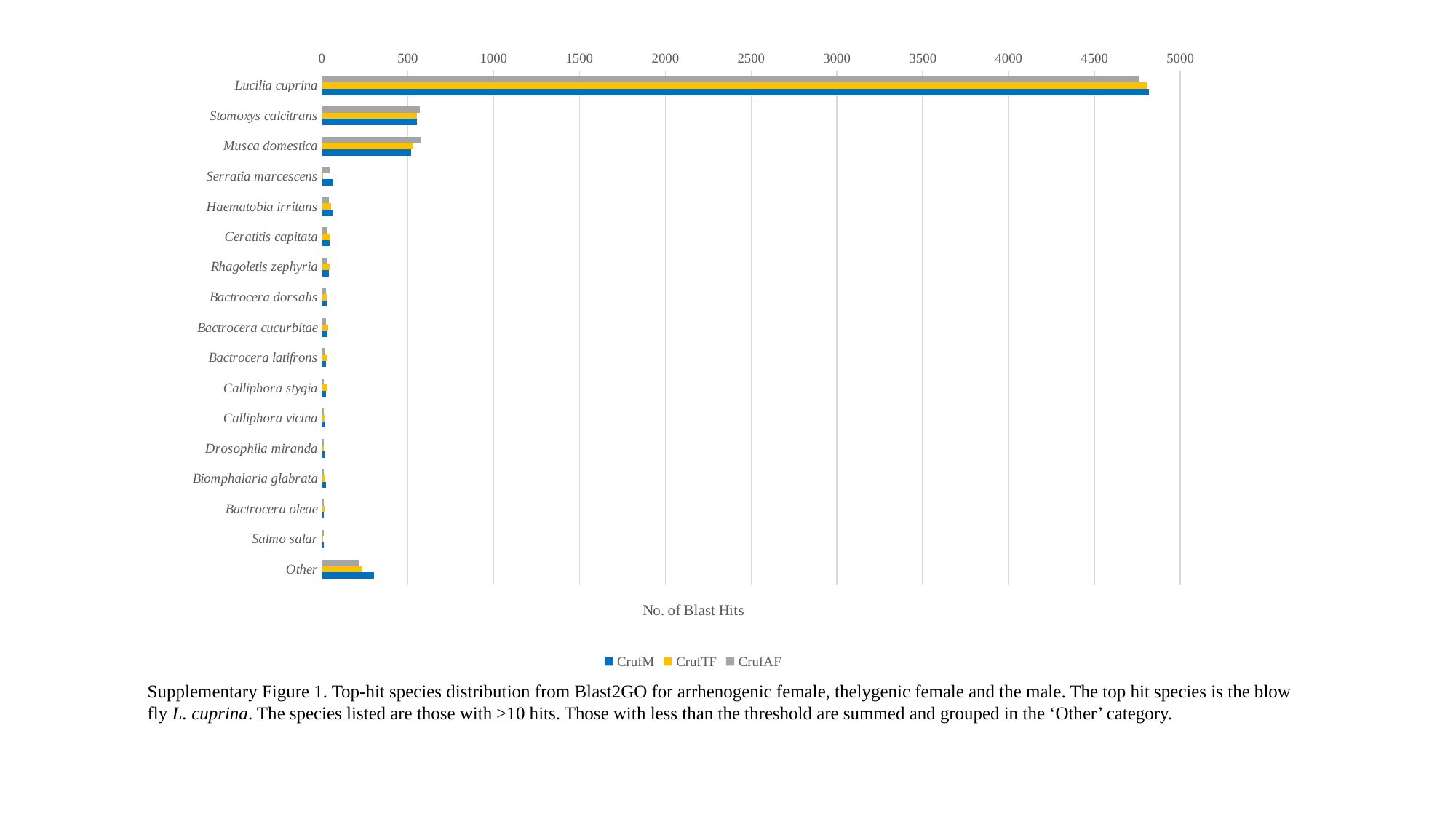

### Chart
| Category | CrufAF | CrufTF | CrufM |
|---|---|---|---|
| Lucilia cuprina | 4756.0 | 4808.0 | 4819.0 |
| Stomoxys calcitrans | 569.0 | 554.0 | 553.0 |
| Musca domestica | 575.0 | 532.0 | 521.0 |
| Serratia marcescens | 48.0 | 1.0 | 67.0 |
| Haematobia irritans | 39.0 | 54.0 | 65.0 |
| Ceratitis capitata | 31.0 | 48.0 | 44.0 |
| Rhagoletis zephyria | 29.0 | 44.0 | 41.0 |
| Bactrocera dorsalis | 22.0 | 27.0 | 28.0 |
| Bactrocera cucurbitae | 22.0 | 37.0 | 34.0 |
| Bactrocera latifrons | 18.0 | 30.0 | 22.0 |
| Calliphora stygia | 12.0 | 34.0 | 23.0 |
| Calliphora vicina | 12.0 | 16.0 | 18.0 |
| Drosophila miranda | 11.0 | 11.0 | 15.0 |
| Biomphalaria glabrata | 10.0 | 19.0 | 22.0 |
| Bactrocera oleae | 9.0 | 15.0 | 11.0 |
| Salmo salar | 9.0 | 7.0 | 12.0 |
| Other | 215.0 | 237.0 | 303.0 |Supplementary Figure 1. Top-hit species distribution from Blast2GO for arrhenogenic female, thelygenic female and the male. The top hit species is the blow fly L. cuprina. The species listed are those with >10 hits. Those with less than the threshold are summed and grouped in the ‘Other’ category.
